# Early-Onset Type 2 Diabetes Mellitus

**DOI:** 10.7759/cureus.17578

**Published:** 2021-08-30

**Authors:** Angel Sunny

**Affiliations:** 1 Department of Pediatrics, Jagadguru Sri Shivarathreeshwara Hospital, Mysuru, IND

**Keywords:** cardiovascular disease, new-onset diabetes, diabetes mellitus type 2, pediatric diabetes, pediatrics rehabilitation, glycated hemoglobin (hba1c), pancreatic beta cells, coronavirus disease (covid-19), cardio vascular disease, child and youth mental health

## Abstract

Globally, the prevalence of chronic, non-communicable diseases is increasing at an alarming rate. Amongst it, Type 2 diabetes mellitus (DM) is becoming more prevalent among young individuals due to obesity and sedentary habits. With the advent of COVID-19, there has been an increasing trend for diabetes and its complications. Here we describe a 13-year-old female girl with polyuria, polydipsia for two months with further assessment leading to a diagnosis of Type 2 DM who is now closely monitored by a pediatric endocrinologist. She remains euglycemic with insulin and lifestyle changes. Early-onset DM is complex and requires multidisciplinary care for preventing complications and comorbidities. Hence, early recognition and management are crucial.

## Introduction

Since the first COVID-19 in China was reported, more than two years have passed. The recent updates from the WHO have data showing the most significant proportionate increase in instances of new COVID-19 registered in regions of the Americas (14%) and Western Pacific region (19%) with a cumulative number of total COVID-19 cases surpassing 200 million [1]. Since the pandemic started, the sporadic onset of type 2 diabetes mellitus (DM) among adolescents has alarmingly increased. Evidence regarding the relationship between pandemic and new-onset type 2 DM is limited. However, most young individuals have exhibited increased BMI and other risk factors such as sedentary lifestyle, family history of DM, African or other ethnic minority groups, and less affluent socioeconomic status [2]. In this case report, we present a 13-year-old female with an early-onset type 2 DM.

## Case presentation

A 13-year-old female presented in a clinic with a one-month history of excessive thirst and excessive urination, causing disturbed sleep. There was no history of polyphagia, weight loss, dysuria. The child is the second of three children of a working-class family. A positive family history of type 2 DM in the maternal grandmother was treated with insulin and oral medications. There was no known history of autoimmune disorders. Urinalysis done at the primary care physician's office showed glucose (2000), mild ketones (5), and leukocytes. To investigate further, she was been referred to the emergency department (ED).

She was alert, active, oriented to time, place, and person at the emergency department. Attention, concentration, memory, verbal and mathematical abilities were intact as well. Further findings were an obese teenage girl (BMI-30kg/m^2^) with signs of acanthosis nigricans over the neck, axilla, and inguinal areas. Systemic examination was essentially normal, and there were no signs of diabetic ketoacidosis. Her HbA1c was 11.0, along with a urinalysis at the ED showing glucosuria with no blood, ketones, or bacteria. Complete blood count and basic metabolic panel were normal. 

The patient was admitted on the same day and started treatment with subcutaneous insulin distributed four times a day with correction of serum glucose via insulin sliding scale pre-breakfast, pre-lunch, pre-dinner, and pre-bedtime. Laboratory investigations were sent for C-peptide, insulin level, insulin antibody, anti-glutamic acid decarboxylase (anti-GAD). The patient and her parents were counseled regarding the elimination of high-calorie beverages such as juices, soda, reduction of foods with a high glycaemic index such as table sugar, ice cream, white bread as well as to encourage consumption of food with a low glycaemic index such as pasta, skimmed milk, sweet potatoes. Alongside, counseling was done on the importance of exercises and the necessity of adhering to medications. The aim was to achieve the expected weight for age. The electrolyte results were within normal ranges with sodium 134mmol/l(128-142mmol/l), potassium 4.4mmol/l (3.4-4.8 mmol/l), bicarbonate 27mmol/l (24-30 mmol/l), urea 3.3 mmol/l (2.4-6.0mmol/l), and creatinine 80mmol/l (60-120mmol/l). Serum cortisol level was normal as well. The autoimmune panel came out negative, and a provisional diagnosis of Type 2 DM was made and referred to a pediatric endocrinologist for further assessment and management. The patient and her mother have been taught the method to assess and administer the medication and diet. A calculated carbohydrate chart has been provided as well. She is responding well to the treatment, her symptoms have improved, she is euglycemic. and on regular follow-up with the pediatric endocrinologist.

## Discussion

The prevalence of type 2 DM in children and adolescents has increased worldwide over the past three decades. This increase has coincided with the obesity epidemic, and minority groups are disproportionately affected [3]. The recent COVID-19 pandemic has inflicted a further increase in this scenario. Alongside the rising trend in individuals affected with type 2 DM in a developing country, female teenagers are more prone to develop these conditions than male counterparts [4]. This is explained because of the insulin resistance secondary to increased growth hormone secretion and sex steroids during the pubertal period [4]^ ^and the dietary influence of westernization. This is also concerning when the individual enters the reproductive period. Pre-pregnancy counseling and contraception are imperative in this age group to offset preventable diabetes-related pregnancy and fetal complications. There is evidence that shows the minority are mostly affected [4]. Bogalusa heart study evaluated plasma glucose and insulin levels during an oral glucose tolerance test (OGTT) in 377 children from five to 17 years of age in a biracial community. After adjusting for age, weight, ponderal index, and pubertal stage, African-Americans showed higher insulin responses than their white counterparts, suggesting a compensated insulin resistance. A different study using the clamp method resulted in a 30% decrease in insulin sensitivity among African-American adolescents compared to white adolescents. Both these studies suggest that minor children may have increased susceptibility to develop type 2 DM, which causes the disease to elicit during physiologic (puberty) or pathologic (obesity) states of insulin resistance [4].

Another interesting finding researchers have demonstrated is that of increased betatrophin levels and their relation with insulin resistance [5]. They are hoping that this could be a potential therapeutic implication for the treatment of diabetes, and investigations are increasing. The authors also showed that overexpression of betatrophin does not increase B-cell production or improve glycemic control. Studies have demonstrated that insulin resistance induces an increase in B-cell response to compensate for the increased insulin demand in normal individuals. Studies using human and mouse models have reported that defects in insulin-signaling pathways in B-cells reduce secretory functions [5]. This explains the pathologic cause of obesity pertinent among the young population. As the pandemic strikes the world for almost two years at the time of this paper and kids are bound to the home resulting in social restrictions, they tend to have difficulty coping with their social, academic, and behavioral growth. An increase in dependence on fast foods and lack of exercise are causing obesity and associated low self-esteem among the growing population.

Insulin resistance is primarily driven by obesity. However, it is not the degree of obesity but rather the distribution of fat. A combination of high intracellular lipid content, increased visceral fat, decreased subcutaneous fat, and ectopic liver fat deposition is most likely to result in glucose imbalance among young individuals and the pediatric population [6].

An interesting finding from various studies has shown the relationship between lipid level and type 2 DM. When compared with the usual onset group, the early-onset type 2 DM patients were more hypertriglyceridemic with higher total cholesterol, higher total cholesterol/high-density lipoprotein (HDL) ratio, higher low-density lipoprotein levels, and lower HDL cholesterol. Interestingly, a randomized control trial had shown evidence that supplementing probiotics could significantly reduce total cholesterol levels but did not regulate LDL cholesterol or HDL cholesterol concentrations [6]. The subgroup analysis showed that using two probiotics is more beneficial than one to decrease total cholesterol and triglycerides concentrations. Furthermore, powder, not liquid, probiotics could reduce the total cholesterol and triglyceride concentrations. This meta-analysis demonstrated that probiotic supplementation helps reduce total cholesterol and triglyceride concentrations in type 2 DM patients. However, more well-controlled trials are needed to clarify the benefits of probiotics on dyslipidemia in type 2 DM patients [6].

Also, early-onset type 2 DM patients showed significantly worse glycemic control and rapid decline in insulin secretion compared with usual onset type 2 DM patients [7]. To reduce the lifetime risk of coronary heart disease, early and aggressive treatment of cardiovascular risk factors in young people with type 2 DM is recommended; however, the evidence of cardioprotective medication use among children is suboptimal. This leads to a higher level of cardiometabolic risk. Using data from 41,000 patients from nine Asian countries showed that those with young-onset type 2 DM had higher mean concentrations of HbA1c and LDL cholesterol leading to mortality at a younger age. In addition, the cardiovascular risk profile and higher relative risk of myocardial and cardiovascular death have been reported among early-onset cohort than in the later-onset [7]^.^ This could result from inadequate management. It emphasizes the need for education and healthcare providers to ensure accessible, patient-centered, coordinated, and continuous effective care during this period. 

Another area to be addressed that has invaluable importance is the psychological aspects of these children. They need to strive for excellent glucose control and body weight and cope with the need to take multiple medications and complications and comorbidities starting early in their life. Those with early-onset type 2 DM require the cooperation of multiple specialties with access to psychological, dietary, and bariatric specialists. There is a growing necessity to develop a curriculum that meets the early onset of DM with a specific focus on pre-conceptual care, body image, self-esteem, and recognizing and managing depression.

The ideal solution would be to prevent type 2 DM in young people. Tackling the increasing trend of obesity worldwide will require combined efforts on behalf of individuals, healthcare professionals, organizations, and legislators to make changes to reduce the prevalence of obesity [6]. Policies on food marketing, exercise, structured environment, transportation, and physical activity in schools and work will need to change to bring out the desired change effectively. In the meantime, there is a need to identify and screen those at risk of type 2 DM so that intensive lifestyle interventions can be delivered to attempt to prevent developing diabetes or other metabolic syndromes. Additionally, the COVID-19 pandemic, lockdown, absence of school activities, and increased screen time have sky-rocketed various health-related issues among children globally. There has been a very sharp rise in the number of type 2 DM among children predisposed to risk factors such as obesity, family history of type 2 DM, and polycystic ovary syndrome [8]. These children often require admission to critical care. Therefore, it is imperative to recognize this potential complication in the pediatric population and provide the required care and follow-up for a better outcome.

## Conclusions

Early-onset type 2 DM is an increasing problem that affects the individual physically, psychologically, and financially. A high index of suspicion is needed in an overweight child with hyperglycemia, family history of DM, female sex, and the African race. These groups would benefit from multidisciplinary interventions to reduce significant morbidity, thereby improving their quality of life.
